# 2-[1-(2-Hy­droxy-4-meth­oxy­phen­yl)ethyl­idene]-*N*-methyl­hydrazinecarbo­thio­amide

**DOI:** 10.1107/S1600536813019831

**Published:** 2013-07-24

**Authors:** Brian J. Anderson, Michael B. Freedman, Sean P. Millikan, Jerry P. Jasinski

**Affiliations:** a Department of Chemistry, Keene State College, 229 Main Street, Keene, NH 03435-2001, USA

## Abstract

In the title compound, C_11_H_15_N_3_O_2_S, the dihedral angle between the mean planes of the benzene ring and hydrazinecarbo­thio­amide group is 9.2 (1)°. An intra­molecular O—H⋯N hydrogen bond is observed, serving to maintain an approximately planar conformation for the molecule. In the crystal, inversion dimers linked by C—H⋯O inter­actions occur. Further C—H⋯O contacts link dimers into (010) chains.

## Related literature

For the synthesis and structure of thio­semicarbazones as ligands, see: Lobana *et al.* (2009 ▶, 2012 ▶). For palladium complexes with thio­semicarbazone ligands, see: Chellan *et al.* (2010 ▶). For related structures, see: Anderson *et al.* (2012 ▶, 2013 ▶). For bond lengths, see: Allen *et al.* (1987 ▶).
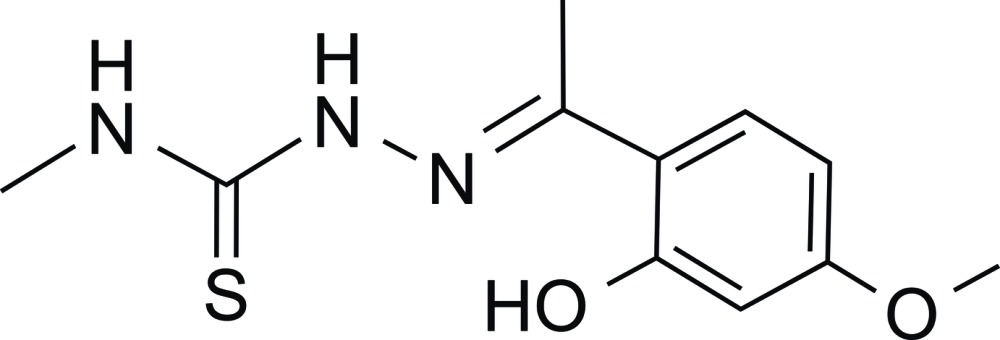


## Experimental

### 

#### Crystal data

C_11_H_15_N_3_O_2_S
*M* *_r_* = 253.32
Monoclinic, 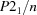

*a* = 10.9881 (8) Å
*b* = 9.1468 (6) Å
*c* = 12.5575 (9) Å
β = 109.400 (8)°
*V* = 1190.45 (15) Å^3^
*Z* = 4
Mo *K*α radiation
μ = 0.27 mm^−1^
*T* = 173 K
0.42 × 0.38 × 0.14 mm

#### Data collection

Agilent Xcalibur (Eos, Gemini) diffractometer
Absorption correction: multi-scan (*CrysAlis PRO* and *CrysAlis RED*; Agilent, 2012 ▶) *T*_min_ = 0.728, *T*_max_ = 1.000
13894 measured reflections
4104 independent reflections
3320 reflections with *I* > 2σ(*I*)
*R*_int_ = 0.043

#### Refinement

*R*[*F* ^2^ > 2σ(*F* ^2^)] = 0.076
*wR*(*F* ^2^) = 0.231
*S* = 1.16
4104 reflections
158 parameters
H-atom parameters constrained
Δρ_max_ = 1.21 e Å^−3^
Δρ_min_ = −0.46 e Å^−3^

### 

Data collection: *CrysAlis PRO* (Agilent, 2012 ▶); cell refinement: *CrysAlis PRO*; data reduction: *CrysAlis RED* (Agilent, 2012 ▶); program(s) used to solve structure: *SUPERFLIP* (Palatinus & Chapuis, 2007 ▶); program(s) used to refine structure: *SHELXL2012* (Sheldrick, 2008 ▶); molecular graphics: *OLEX2* (Dolomanov *et al.*, 2009 ▶); software used to prepare material for publication: *OLEX2*.

## Supplementary Material

Crystal structure: contains datablock(s) global, I. DOI: 10.1107/S1600536813019831/hg5331sup1.cif

Structure factors: contains datablock(s) I. DOI: 10.1107/S1600536813019831/hg5331Isup2.hkl

Supplementary material file. DOI: 10.1107/S1600536813019831/hg5331Isup3.cml

Additional supplementary materials:  crystallographic information; 3D view; checkCIF report

## Figures and Tables

**Table 1:** Hydrogen-bond geometry (Å, °)

*D*—H⋯*A*	*D*—H	H⋯*A*	*D*⋯*A*	*D*—H⋯*A*
O1—H1⋯N1	0.82	1.85	2.566 (3)	145
C10—H10*A*⋯O2^i^	0.96	2.59	3.301 (4)	132
C10—H10*C*⋯O1^ii^	0.96	2.57	3.481 (4)	158

Symmetry codes: (i) 
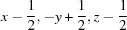
; (ii) 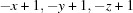.

**Fig. 1.**
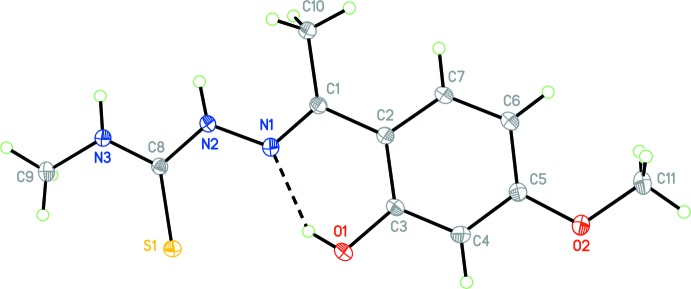
ORTEP drawing of (I) showing the atom labeling scheme and 30% probability displacement ellipsoids. Dashed lines indicate O1—H1···N1 intramolecular hydrogen bonds.

**Fig. 2.**
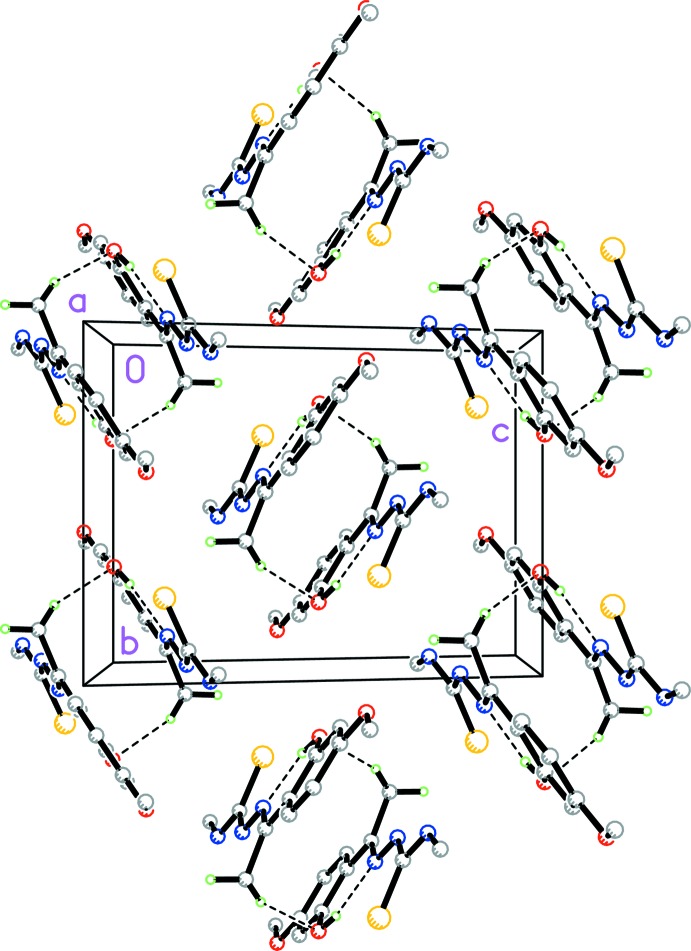
Molecular packing for (I) viewed along the b axis. Dashed lines indicate O—H···N intramolecular hydrogen bonds and weak C—H···O intermolecular interactions linking the molecules into dimers along (010).

**Fig. 3.**
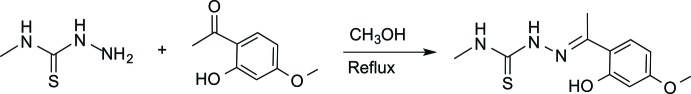
Synthesis of (I).

## supplementary crystallographic information

### Comment

Thiosemicarbazones are a versatile class of ligands that can adopt multiple modes of binding to a metal (Lobana, *et al.*, 2009) and the synthesis and structure determination of these metal complexes is an active area of research. (Lobana, *et al.*, 2012) Palladium complexes with thiosemicarbazone ligands have been shown to have a variety of biological activity including anti-fungal and anti-tumor activity. (Chellan, *et al.*, 2010). We have previously reported the structure of two analogous novel thiosemicarbazones (Anderson, *et al.*, 2012; Anderson, *et al.*, 2013). Here, we report the synthesis and crystal structure of a novel thiosemicarbazone ligand, (I), C_11_H_15_N_3_O_2_S .

In (I), the dihedral angle between the mean planes of the benzene ring and hydrazinecarbothioamide group (N1/N2/C8/S1/N3) is 9.2 (1)° (Fig. 1). Bond lengths are in normal ranges (Allen *et al.*, 1987). In the crystal, an intramolecular O—H···N hydrogen bond is observed serving to keep the molecule in a nearly planar conformation. Additional weak and C—H···O intermolecular interactions (Table 1) assist in linking the molecules into dimers along (010) and influence crystal packing (Fig. 2).

### Experimental

A 50 mL round bottom flask was charged with 0.218 g (1.31 mmol) of 2'-hydroxy-4'-methoxyacetophenone, 0.138 g (1.31 mmol) of 4-methyl-3- thiosemicarbazide, dissolved in 20 mL of methanol. The resulting colorless solution was refluxed for 48 hours and then a drop of concentrated HCl was added and the solution was refluxed for an additional 48 hours. The resulting yellow solution was transferred to a 125 mL separatory funnel. Dichloromethane (10 mL) and water (10 mL) were added, and the organic layer was separated. The aqueous layer was extracted with an additional 10 mL of dichloromethane. The organic layers were combined, washed with brine (2 x 10 mL), dried with magnesium sulfate, and the solvent was removed in vacuo to give a yellow solid (Fig. 3). The solid was dissolved in hot acetonitrile, allowed to cool to room temperature and then stored at 273 K resulting in colorless crystals (58 mg, 18%) after 24 hours. M.p. 448-453 K.

### Refinement

All of the H atoms were placed in their calculated positions and then refined using the riding model with atom—H lengths of 0.93Å (CH), 0.96Å (CH_3_), 0.86Å (NH) or 0.82Å (OH). Isotropic displacement parameters for these atoms were set to 1.2 (CH, NH) or 1.5 (CH_3_, OH) times *U*_eq_ of the parent atom. Idealised Me refined as rotating group: C9(H9A,H9B,H9C), C10(H10A,H10B,H10C), C11(H11A,H11B,H11C). Idealised tetrahedral OH refined as rotating group: O1(H1).

### Crystal data

**Table:**

C_11_H_15_N_3_O_2_S	*F*(000) = 536
*M**_r_* = 253.32	*D*_x_ = 1.413 Mg m^−^^3^
Monoclinic, *P*2_1_/*n*	Mo *K*α radiation, λ = 0.7107 Å
*a* = 10.9881 (8) Å	Cell parameters from 4367 reflections
*b* = 9.1468 (6) Å	θ = 3.0–32.9°
*c* = 12.5575 (9) Å	µ = 0.27 mm^−^^1^
β = 109.400 (8)°	*T* = 173 K
*V* = 1190.45 (15) Å^3^	Irregular, colourless
*Z* = 4	0.42 × 0.38 × 0.14 mm

### Data collection

**Table:**

Agilent Xcalibur (Eos, Gemini) diffractometer	4104 independent reflections
Radiation source: Enhance (Mo) X-ray Source	3320 reflections with *I* > 2σ(*I*)
Graphite monochromator	*R*_int_ = 0.043
Detector resolution: 16.0416 pixels mm^-1^	θ_max_ = 33.0°, θ_min_ = 3.0°
ω scans	*h* = −15→16
Absorption correction: multi-scan (*CrysAlis PRO* and *CrysAlis RED*; Agilent, 2012)	*k* = −12→13
*T*_min_ = 0.728, *T*_max_ = 1.000	*l* = −18→18
13894 measured reflections	

### Refinement

**Table:**

Refinement on *F*^2^	Primary atom site location: structure-invariant direct methods
Least-squares matrix: full	Hydrogen site location: inferred from neighbouring sites
*R*[*F*^2^ > 2σ(*F*^2^)] = 0.076	H-atom parameters constrained
*wR*(*F*^2^) = 0.231	*w* = 1/[σ^2^(*F*_o_^2^) + (0.0907*P*)^2^ + 2.429*P*] where *P* = (*F*_o_^2^ + 2*F*_c_^2^)/3
*S* = 1.16	(Δ/σ)_max_ < 0.001
4104 reflections	Δρ_max_ = 1.21 e Å^−^^3^
158 parameters	Δρ_min_ = −0.46 e Å^−^^3^
0 restraints	

### Special details

**Table:**

Experimental. ^1^H NMR [(CD_3_)_2_CO]: 11.7 (br s, 1H, NH) 9.52 (br s, 1H, OH) 7.73 (br s, 1H, NH) 7.5 (d, J = 8.6, 1H Ar) 6.47 (d, J = 8.6 1H Ar) 6.42 (d, 1H Ar) 3.80 (s, 3H, CH_3_) 3.13 (d, J = 4.7, 3H, CH_3_) 2.43 (s, 3H, CH_3_)
Geometry. All esds (except the esd in the dihedral angle between two l.s. planes) are estimated using the full covariance matrix. The cell esds are taken into account individually in the estimation of esds in distances, angles and torsion angles; correlations between esds in cell parameters are only used when they are defined by crystal symmetry. An approximate (isotropic) treatment of cell esds is used for estimating esds involving l.s. planes.

### Fractional atomic coordinates and isotropic or equivalent isotropic displacement parameters (Å^2^)

**Table:**

	*x*	*y*	*z*	*U*_iso_*/*U*_eq_	
S1	0.21259 (6)	0.28834 (7)	0.36426 (6)	0.02286 (19)	
O1	0.55069 (19)	0.2003 (2)	0.50127 (19)	0.0280 (4)	
H1	0.4936	0.2530	0.4608	0.042*	
O2	0.9881 (2)	0.0943 (3)	0.60376 (19)	0.0309 (5)	
N1	0.4600 (2)	0.4175 (3)	0.36942 (19)	0.0220 (4)	
N2	0.3540 (2)	0.5019 (3)	0.31878 (19)	0.0220 (4)	
H2	0.3628	0.5869	0.2929	0.026*	
N3	0.1393 (2)	0.5407 (3)	0.2551 (2)	0.0240 (4)	
H3	0.1595	0.6225	0.2317	0.029*	
C1	0.5729 (2)	0.4652 (3)	0.3762 (2)	0.0198 (4)	
C2	0.6806 (2)	0.3678 (3)	0.4335 (2)	0.0201 (5)	
C3	0.6655 (2)	0.2410 (3)	0.4935 (2)	0.0207 (5)	
C4	0.7702 (3)	0.1533 (3)	0.5478 (2)	0.0245 (5)	
H4	0.7585	0.0703	0.5862	0.029*	
C5	0.8923 (3)	0.1875 (3)	0.5459 (2)	0.0232 (5)	
C6	0.9109 (3)	0.3099 (3)	0.4871 (2)	0.0260 (5)	
H6	0.9923	0.3327	0.4844	0.031*	
C7	0.8055 (3)	0.3967 (3)	0.4329 (2)	0.0250 (5)	
H7	0.8182	0.4786	0.3939	0.030*	
C8	0.2350 (2)	0.4514 (3)	0.3095 (2)	0.0192 (4)	
C9	0.0037 (3)	0.5110 (4)	0.2322 (3)	0.0299 (6)	
H9A	−0.0070	0.4535	0.2924	0.045*	
H9B	−0.0298	0.4582	0.1624	0.045*	
H9C	−0.0422	0.6016	0.2264	0.045*	
C10	0.5946 (3)	0.6111 (3)	0.3322 (3)	0.0255 (5)	
H10A	0.5542	0.6133	0.2517	0.038*	
H10B	0.6856	0.6278	0.3508	0.038*	
H10C	0.5580	0.6860	0.3658	0.038*	
C11	1.1161 (3)	0.1303 (4)	0.6095 (3)	0.0349 (7)	
H11A	1.1420	0.2195	0.6510	0.052*	
H11B	1.1191	0.1425	0.5344	0.052*	
H11C	1.1736	0.0529	0.6467	0.052*	

### Atomic displacement parameters (Å^2^)

**Table:**

	*U*^11^	*U*^22^	*U*^33^	*U*^12^	*U*^13^	*U*^23^
S1	0.0232 (3)	0.0190 (3)	0.0279 (3)	−0.0010 (2)	0.0104 (2)	0.0013 (2)
O1	0.0214 (9)	0.0276 (10)	0.0383 (11)	−0.0008 (8)	0.0143 (8)	0.0071 (8)
O2	0.0216 (9)	0.0354 (11)	0.0358 (11)	0.0056 (8)	0.0094 (8)	0.0128 (9)
N1	0.0192 (10)	0.0224 (10)	0.0245 (10)	0.0012 (8)	0.0072 (8)	0.0009 (8)
N2	0.0172 (9)	0.0207 (10)	0.0287 (11)	−0.0001 (8)	0.0083 (8)	0.0027 (8)
N3	0.0197 (10)	0.0212 (10)	0.0320 (11)	0.0019 (8)	0.0099 (8)	0.0049 (8)
C1	0.0193 (11)	0.0200 (11)	0.0199 (10)	−0.0012 (8)	0.0063 (8)	−0.0006 (8)
C2	0.0193 (11)	0.0198 (10)	0.0212 (10)	−0.0014 (9)	0.0068 (8)	−0.0001 (8)
C3	0.0203 (11)	0.0217 (11)	0.0221 (11)	−0.0022 (9)	0.0098 (9)	−0.0007 (9)
C4	0.0262 (12)	0.0228 (12)	0.0266 (12)	−0.0002 (10)	0.0115 (10)	0.0047 (9)
C5	0.0209 (11)	0.0251 (12)	0.0239 (11)	0.0009 (9)	0.0076 (9)	0.0021 (9)
C6	0.0197 (11)	0.0277 (13)	0.0314 (13)	−0.0024 (10)	0.0096 (10)	0.0042 (10)
C7	0.0205 (11)	0.0247 (12)	0.0308 (13)	−0.0024 (9)	0.0101 (10)	0.0044 (10)
C8	0.0200 (11)	0.0191 (10)	0.0200 (10)	−0.0002 (8)	0.0086 (8)	−0.0020 (8)
C9	0.0203 (12)	0.0316 (14)	0.0383 (15)	0.0048 (10)	0.0103 (11)	0.0067 (12)
C10	0.0200 (11)	0.0207 (11)	0.0348 (14)	−0.0013 (9)	0.0080 (10)	0.0026 (10)
C11	0.0204 (12)	0.0417 (17)	0.0424 (17)	0.0044 (12)	0.0104 (11)	0.0084 (14)

### Geometric parameters (Å, º)

**Table:**

S1—C8	1.695 (3)	C3—C4	1.383 (4)
O1—H1	0.8200	C4—H4	0.9300
O1—C3	1.350 (3)	C4—C5	1.386 (4)
O2—C5	1.362 (3)	C5—C6	1.393 (4)
O2—C11	1.423 (4)	C6—H6	0.9300
N1—N2	1.366 (3)	C6—C7	1.383 (4)
N1—C1	1.290 (3)	C7—H7	0.9300
N2—H2	0.8600	C9—H9A	0.9600
N2—C8	1.354 (3)	C9—H9B	0.9600
N3—H3	0.8600	C9—H9C	0.9600
N3—C8	1.328 (3)	C10—H10A	0.9600
N3—C9	1.446 (4)	C10—H10B	0.9600
C1—C2	1.465 (4)	C10—H10C	0.9600
C1—C10	1.494 (4)	C11—H11A	0.9600
C2—C3	1.422 (4)	C11—H11B	0.9600
C2—C7	1.400 (4)	C11—H11C	0.9600
			
C3—O1—H1	109.5	C7—C6—H6	120.8
C5—O2—C11	117.3 (2)	C2—C7—H7	118.3
C1—N1—N2	119.4 (2)	C6—C7—C2	123.4 (2)
N1—N2—H2	120.1	C6—C7—H7	118.3
C8—N2—N1	119.8 (2)	N2—C8—S1	122.13 (19)
C8—N2—H2	120.1	N3—C8—S1	123.55 (19)
C8—N3—H3	117.4	N3—C8—N2	114.3 (2)
C8—N3—C9	125.1 (2)	N3—C9—H9A	109.5
C9—N3—H3	117.4	N3—C9—H9B	109.5
N1—C1—C2	115.4 (2)	N3—C9—H9C	109.5
N1—C1—C10	123.1 (2)	H9A—C9—H9B	109.5
C2—C1—C10	121.5 (2)	H9A—C9—H9C	109.5
C3—C2—C1	122.5 (2)	H9B—C9—H9C	109.5
C7—C2—C1	121.1 (2)	C1—C10—H10A	109.5
C7—C2—C3	116.4 (2)	C1—C10—H10B	109.5
O1—C3—C2	122.7 (2)	C1—C10—H10C	109.5
O1—C3—C4	116.6 (2)	H10A—C10—H10B	109.5
C4—C3—C2	120.7 (2)	H10A—C10—H10C	109.5
C3—C4—H4	119.6	H10B—C10—H10C	109.5
C3—C4—C5	120.8 (2)	O2—C11—H11A	109.5
C5—C4—H4	119.6	O2—C11—H11B	109.5
O2—C5—C4	115.5 (2)	O2—C11—H11C	109.5
O2—C5—C6	124.2 (2)	H11A—C11—H11B	109.5
C4—C5—C6	120.3 (2)	H11A—C11—H11C	109.5
C5—C6—H6	120.8	H11B—C11—H11C	109.5
C7—C6—C5	118.5 (2)		
			
O1—C3—C4—C5	−179.1 (3)	C3—C2—C7—C6	−0.4 (4)
O2—C5—C6—C7	−179.2 (3)	C3—C4—C5—O2	179.1 (2)
N1—N2—C8—S1	2.8 (3)	C3—C4—C5—C6	−1.2 (4)
N1—N2—C8—N3	−177.6 (2)	C4—C5—C6—C7	1.0 (4)
N1—C1—C2—C3	−9.8 (4)	C5—C6—C7—C2	−0.2 (4)
N1—C1—C2—C7	171.0 (2)	C7—C2—C3—O1	179.8 (2)
N2—N1—C1—C2	179.1 (2)	C7—C2—C3—C4	0.3 (4)
N2—N1—C1—C10	0.7 (4)	C9—N3—C8—S1	−1.7 (4)
C1—N1—N2—C8	178.8 (2)	C9—N3—C8—N2	178.7 (3)
C1—C2—C3—O1	0.6 (4)	C10—C1—C2—C3	168.6 (2)
C1—C2—C3—C4	−179.0 (2)	C10—C1—C2—C7	−10.6 (4)
C1—C2—C7—C6	178.9 (3)	C11—O2—C5—C4	−176.4 (3)
C2—C3—C4—C5	0.5 (4)	C11—O2—C5—C6	3.8 (4)

### Hydrogen-bond geometry (Å, º)

**Table:**

*D*—H···*A*	*D*—H	H···*A*	*D*···*A*	*D*—H···*A*
O1—H1···N1	0.82	1.85	2.566 (3)	145
C10—H10*A*···O2^i^	0.96	2.59	3.301 (4)	132
C10—H10*C*···O1^ii^	0.96	2.57	3.481 (4)	158

Symmetry codes: (i) *x*−1/2, −*y*+1/2, *z*−1/2; (ii) −*x*+1, −*y*+1, −*z*+1.
